# Synthesis of Poly(*N*-vinylpyrrolidone)-Based Polymer Bottlebrushes by ATRPA and RAFT Polymerization: Toward Drug Delivery Application

**DOI:** 10.3390/polym11061079

**Published:** 2019-06-22

**Authors:** Yi-Shen Huang, Jem-Kun Chen, Shiao-Wei Kuo, Ya-An Hsieh, Shota Yamamoto, Jun Nakanishi, Chih-Feng Huang

**Affiliations:** 1 Department of Chemical Engineering, National Chung Hsing University, 145 Xingda Road, Taichung 40227, Taiwan; yishen617@gmail.com (Y.-S.H.); yaan_hsieh@yahoo.com.tw (Y.-A.H.); 2 Department of Materials Science and Engineering, National Taiwan University of Science and Technology, Taipei 10607, Taiwan; jkchen@mail.ntust.edu.tw; 3 Department of Materials and Optoelectronic Science, Center of Crystal Research, National Sun Yat-Sen University, Kaohsiung 804, Taiwan; kuosw@faculty.nsysu.edu.tw; 4 Department of Medicinal and Applied Chemistry, Kaohsiung Medical University, Kaohsiung 807, Taiwan; 5 World Premier International Research Center for Materials Nanoarchitectonics (WPI-MANA), National Institute for Materials Science (NIMS), 1-1, Namiki, Tsukuba, Ibaraki 305-0044, Japan; YAMAMOTO.Shota@nims.go.jp

**Keywords:** atom transfer radical polyaddition (ATRPA), reversible addition–fragmentation chain transfer (RAFT) polymerization, functional polyesters, poly(*N*-vinylpyrrolidone), amphiphilic polymer bottlebrush

## Abstract

Atom transfer radical polyaddition (ATRPA) was utilized herein to synthesize a specific functional polyester. We conducted ATRPA of 4-vinylbenzyl 2-bromo-2-phenylacetate (VBBPA) inimer and successfully obtained a linear type poly(VBBPA) (PVBBPA) polyester with benzylic bromides along the backbone. To obtain a novel amphiphilic polymer bottlebrush, however, the lateral ATRP chain extension of PVBBPA with *N*-vinyl pyrrolidone (NVP) met the problem of quantitative dimerization. By replacing the bromides to xanthate moieties efficiently, we thus observed a pseudo linear first order reversible addition–fragmentation chain transfer (RAFT) polymerization to obtain novel poly(4-vinylbenzyl-2-phenylacetate)-*g*-poly(NVP) (PVBPA-*g*-PNVP) amphiphilic polymer bottlebrushes. The critical micelle concentration (CMC) and particle size of the amphiphilic polymer bottlebrushes were characterized by fluorescence spectroscopy, dynamic light scattering (DLS), and scanning electron microscopy (SEM) (CMCs < 0.5 mg/mL; particle sizes = ca. 100 nm). Toward drug delivery application, we examined release profiles using a model drug of Nile red at different pH environments (3, 5, and 7). Eventually, low cytotoxicity and well cell uptake of the Madin-Darby Canine Kidney Epithelial (MDCK) for the polymer bottlebrush micelles were demonstrated.

## 1. Introduction

With the developments of living polymerizations, we can polymerize a variety of monomers to obtain functional polymers with controllable molecular weights (MWs), low polydispersity (PDI), sequence-controlled macromolecules [1], and various topologies [2,3,4,5,6,7,8]. Reversible-deactivation radical polymerizations (RDRPs) are one of the most common-used tools in living polymerizations [9,10]. RDRPs generally include nitroxide-mediated radical polymerization (NMRP) [11,12,13], reversible addition-fragmentation chain transfer (RAFT) polymerization [14,15,16], atom transfer radical polymerization (ATRP)] [17,18,19], and ring-opening (metathesis) polymerization (RO(M)P) [20,21,22]. 

Aliphatic polyesters, involving polylactides, polyglycolides, poly(ε-caprolactone)s and so on, have been investigated extensively due to their enormous bio-related applications, such as tissue engineering and drug/gene delivery therapy. In general, aliphatic polyesters can be synthesized by diacid (AA) and diol (BB) monomers or hydroxyl-acid (AB) monomers. Besides, ring-opening polymerizations (ROPs) of lactones have also been commonly employed [23,24,25,26,27]. Through these methods, however, introduction of functionalities on the polyester backbones is still challenging and limited. Therefore, some other intriguing polymerization techniques, extended from organic reactions with high atom efficiency to synthesize functional aliphatic polyesters, have been investigated [28,29,30,31,32,33,34,35,36,37,38,39]. In addition, radical polymerizations can also be applied to produce degradable polymers which might be similar or mimic the properties of polyesters [40]. For instance, polymer backbones containing cleavable linkages can be accomplished by conventional free radical copolymerization of common monomers and cyclic ketene [41,42]. Using RDRPs, introduction of cleavable functional groups can be easily attained through copolymerization with specific monomers. Thus, radical ring-opening polymerizations (RROPs) of cyclic monomers have been demonstrated as an exciting method for the synthesis of aliphatic polyesters which can introduce a variety of functionality in the backbones [43,44,45,46]. However, functional monomers that can be conducted with RROP are still limited. On the other hand, poly(*N*-vinylpyrrolidone) (PNVP) with the nature of water-soluble, biocompatibility, and low toxicity has also been widely employed in bio-related applications. To the best of our knowledge, synthesis of polyester as a hydrophobic backbone grafted with PNVP as a hydrophilic brush is still challenging. The novel amphiphilic polymer bottlebrush could facilely form micelles and we could expect that the novel PNVP-based amphiphilic polymer bottlebrushes might have potential in bio-related applications. 

Extended from atom transfer radical polymerization (ATRP) [19,47,48,49,50], atom transfer radical polyaddition (ATRPA) [51,52,53] provides an alternative approach to the synthesis of aliphatic polyesters. Kamigaito and co-workers demonstrated the first perfect metal-catalyzed radical polyaddition by fully suppressing the formation of branched structures in several days. The obtained linear type aliphatic polyesters contain numerous functionalizable chlorines along the polyester backbone [51]. They further synthesized sequence-regulated vinyl polymers using a similar concept by designing AB monomers or AA and BB monomers [54]. Li and co-workers studied the manipulations of atom transfer self-condensing vinyl polymerization (ATSCVP), conventional free radical polymerization, and ATRPA to precisely control polymer topology from hyperbranched to linear polymers [55]. Notably, Li and co-workers conducted polyadditions of an AB inimer or AA/BB monomer pairs containing a styrenic structure (i.e., A) and bromoisobutyryl-type initiating site (i.e., B). The rate of polyaddition was significantly improved due to the high reactivity between the bromoisobutyryl-type initiating site and styrene structure in the presence of a copper catalyst. Meanwhile, the newly formed initiating site (i.e., benzyl halides) along the backbone cannot be re-activated due to its low activation rate constant at low temperature (i.e., 0 °C). Namely, a large difference in initiation rate between chain end initiating site (i.e., bromoisobutyryl) and backbone initiating site (i.e., benzyl halide) attained high selectivity to obtain a linear type polyester. Thus, an effective pair of A and B functional groups plays a crucial role in achieving perfect ATRPA.

In our previous study, we demonstrated a highly efficient ATRPA of AB type inimer containing an ethyl 2-bromo-2-phenylacetate (EBPA) based initiating site and a styrene group [56]. Accordingly, our previously reported 4-vinylbenzyl 2-bromo-2-phenylacetate (VBBPA) inimer possesses very high activation rate of the EBPA initiating site at the polyester chain end and high selectivity for the initiations between the chain end and the polyester backbone (i.e., benzyl halide initiating sites). Based on the mechanism study, the copper catalysts possess high selectivity between the initiating site at chain end (EBPA) and the initiating site at backbone (PEBr) (*k*_act,EBPA_/*k*_act,PEBr_ = ca. 30,000) [56]. Therefore, we attained fast ATRPA and obtain linear type polyesters. Different from the traditional polyesters (e.g., PLA, PLGA, PCL), the resulted polyesters possess numerous of functionalizable sites and retain the degradable property. In this study, we performed ATRPA of VBBPA and employed ATRP or RAFT polymerization to prepare a novel PNVP-based amphiphilic polymer bottlebrush. We discussed the synthetic results in detail. The novel amphiphilic polymer bottlebrush was then conducted micellization and characterized by using fluorescence spectroscopy, dynamic light scattering (DLS), and scanning electron microscopy (SEM). We performed the drug release behaviors using a model drug of Nile red. Toward the application of drug delivery, we examined cytotoxicity and cell uptake of the Madin-Darby Canine Kidney Epithelial (MDCK) in the presence of the amphiphilic polymer bottlebrush micelles. We designed an alternative strategy to the preparations of amphiphilic polymer bottlebrush for the endocytosis of MDCK cells.

## 2. Materials and Methods 

### 2.1. Materials 

4-Vinylbenzyl chloride (VBC, 90%), 4,4’-dinonyl-2,2’-bipyridine (dNbpy, 97%), *N,N,N′,N′′,N′′*-pentamethyldiethylenetriamineethanol (PMDETA, 99%), chloroform (99%), anisole (99%), methanol (99.9%), *N,N*-dimethylformamide (DMF, 99.5%), diethyl ether (99.9%), ethyl acetate (EA, 100%), n-hexane (95%), ), pyrene (97%), and potassium ethyl xanthate (PEX, 97%) were purchased from Sigma–Aldrich (St. Louis, MO, US) and used without purification. Synthesis of 4-vinylbenzyl 2-bromo-2-phenylacetate (VBBPA) was according to previous literature [56]. Tetrahydrofuran (THF, 99%) and dichloromethane (DCM, 99.9%) were distilled and stored in molecular sieve prior to use. Madin-Darby Canine Kidney (MDCK) epithelial cells were grown at pH 7.3 in DMEM high glucose medium containing 5% fetal bovine serum (FBS) and 10 mM HEPES-KOH at 37 °C with an atmosphere inlet of ca. 5% CO_2_. CellTiter 96^®^ AQueous Non-Radioactive Cell Proliferation Assay (g5421) was used to stain the cells.

### 2.2. Synthesis of Poly(4-vinylbenzyl 2-bromo-2-phenylacetate) (PVBBPA) by ATRPA 

An example: a ratio of reagents was VBBPA/CuBr_2_/Cu/dNbpy = 50/2/1/6 in anisole ([VBBPA]_0_ = 1.8 M). VBBPA, CuBr_2_, dNbpy and anisole were added to a 10 mL Schlenk flask. The mixture was deoxygenated by three freeze–pump–thaw cycles. One more freeze–pump–thaw cycle was conducted after adding copper powder into the flask and backfilled with nitrogen. An initial sample was taken via a syringe and the flask was kept at 40 °C. After the desired time, the reaction was stopped by placing the flask in an ice bath and the contents were exposed to air. The mixture was diluted by THF, and passed through the neutral Al_2_O_3_ column to remove the copper complexes. THF was removed under vacuum. The residue was precipitated twice in petroleum ether to obtain white powders of PVBBPA (yield: 57.5%; *M*_n_ = 11,200, PDI (*M*_w_/*M*_n_) = 1.58). ^1^H NMR (400 MHz, CDCl_3_, δ = ppm): 7.2 (m, 9H), 5.1 (m, 2H), 4.6 (m, 1H), 3.9 (m, 1H), 2.27 (m, 2H).

### 2.3. Synthesis of Poly(4-vinylbenzyl-2-(ethyl xanthate)-2-phenylacetate) (PVBXPA) Macro-CTA

PVBBPA (13.4 g, 3 mmol) and PEX (13.4 g, 40 mmol) were dissolved in acetone (40 mL) stirring at room temperature (RT) for 24 h. The solution was condensed by a rotary and the residue was dissolved in DCM (60 mL). The impurities were extracted by brine and de-ionized (DI) water twice. The solution was precipitated in petroleum ether to obtain PVBXPA macro-chain transfer agent (macro-CTA) (yield: 77.5%; *M*_n_ = 12,600, PDI = 1.60). ^1^H NMR (400 MHz, CDCl_3_, δ = ppm): 7.09 (m, 9H), 4.99 (m, 2H), 4.5 (m, 1H), 3.5 (m, 1H), 2.27 (m, 2H), 1.62 (m, 3H), 1.26 (m, 2H).

### 2.4. Synthesis of PVBPA-g-PNVP Amphiphilic Polymer Bottlebrush by ATRP or RAFT Polymerization

Chain extension by ATRP: a ratio of reagents was NVP/PVBBPA/CuBr/PMDETA = 200/1/1/1 in anisole (PVBBPA: *M*_n_ = 11,200 and PDI = 1.58; [NVP]_0_ = 4.0 M). PVBBPA, NVP, PMDETA, and anisole were added to a 10 mL Schlenk flask and deoxygenated by three freeze–pump–thaw cycles. In a frozen state, CuBr was added. The flask was deoxygenated by additional two freeze–pump–thaw cycles and backfilled with nitrogen, and an initial sample was taken via a syringe. An initial sample was taken and then the solution was stirred at 80 °C to carry out ATRP chain extension. The NVP conversion was measured by gas chromatograph (GC). After the reaction completed, the flask was quenched in an ice bath and the contents were exposed to air. The mixture was then passed through an alumina column and concentrated to obtain the crude product.

Chain extension by RAFT polymerization: a molar equivalent ratio of reagents was NVP/PVBXPA/AIBN = 20/1/0.1 in anisole (PVBXPA: *M*_n_ = 12600 and PDI = 1.60; [NVP]_0_ = 4.0 M). PVBXPA, NVP, AIBN, and anisole were added to a 10 mL Schlenk flask. The mixture was deoxygenated by means of three freeze–pump–thaw cycles. The flask was backfilled with nitrogen, and an initial sample was taken via a syringe. The flask was kept at 60 °C to carry out RAFT polymerization. The reaction was stopped by placing the flask in an ice bath, exposing the mixture to air, diluting by THF, and precipitated in n-hexane. A white solid was obtained and dried under reduced pressure to obtained PNVP-based amphiphilic polymer bottlebrush (yield: 47.5%; PVBPA_34_-g-PNVP_60_). After grafted PNVP, notably, very weak intensities of GPC traces were observed due to the small refractive index differences between PNVP copolymer and THF eluent. Thus, the repeating units of NVP were estimated from ^1^H NMR spectra.

### 2.5. Encapsulation and Release of Nile Red from PVBPA-g-PNVP Micelles and Tests of MDCK Cell Uptake

A mixture of 20 mg of the PVBPA_34_-g-PNVP_60_ amphiphilic polymer bottlebrush, 2 mg of Nile red, and 1 mL of DMF were stirred in a vial for 1 h. The polymer solution was slowly added to 4 mL of deionized water with stirring for 12 h and dialyzed in a 50 mM PBS solution at pH = 7 for 24 h to remove free Nile red in the solution. The neutral solutions were separately kept in dialysis bags and immersed in PBS solution with different pH (7, 5, and 3). The encapsulation efficiency of Nile red was approximately 70% [57]. The release of Nile red was traced by fluorescence spectroscopy with an excitation wavelength of 560 nm and the observed emission intensity at 645 nm.

The MDCK cells were transferred to a 24-well culture dish and kept as 5000 cells per well. After the cells attached to the bottom of the well, a micelle solution of PVBPA_34_-g-PNVP_60_ amphiphilic polymer bottlebrush (4 mg/mL) containing Nile red was added. After a period of time, the suspension was removed. Cell images were both taken under bright field and excitation light (560 nm) to observe the emission intensity at 645 nm to monitor the Nile red uptake.

### 2.6. Characterization. 

Prior to FT-IR measurement, the sample solutions were cast onto a KBr disk and dried under vacuum. FT-IR spectra were measured by a Perkin Elmer 100 FT-IR Spectrometer (48 scans and a resolution of 1 cm^−1^). ^1^H NMR measurements were performed using a Varian Inova 400 NMR. The the chemical shift of CDCl_3_ was set as 7.26 ppm for calibration. Conversions of NVP were monitored using a HP 5890 GC equipped with an FID detector with a CNW CD-5 column (30 m). The solvent anisole was used as an internal standard. A gel permeation chromatography (GPC) system comprised of a Waters 515 pump, a Waters 410 RI detector, and PSS SDV columns (Linear S and 100 Å pore size) was used to obtain *M*_n_, *M*_w_, and PDI (i.e., *M*_w_/*M*_n_) at 40 °C in THF (flow rate: 1 mL/min). Polystyrene standards were used to establish a calibration line. Different concentrations of polymer solution_(aq)_ were mixed with the same volume of pyrene-containing solution_(aq)_ with a concentration of 4 × 10^−7^ M. Before detecting the critical micelle concentration (CMC), we filtrated the mixtures using a 0.22 μm filter into a vial. CMC measurements were performed using a JASCO FP8500 fluorescence spectroscopy scanned in 300–500 nm (λ_ex_ = 250 nm). Particle sizes were measured by dynamic light scattering (DLS: Brookhaven NanoBrook Zeta PALS instrument). Prior to DLS analysis, the mixture was filtrated through a filter (size of porous PTFE: 0.45 μm; solution concentration: 1 mg mL^–1^).

## 3. Results and Discussion

### 3.1. Kinetic Study of the Synthesis of Amphiphilic Polymer Bottlebrush

**Scheme 1:**
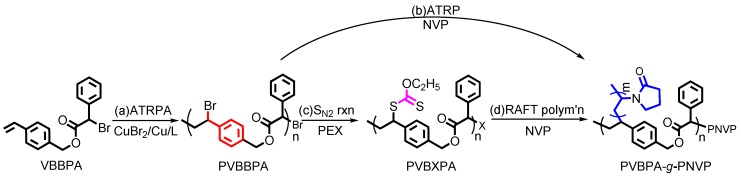
Synthetic routes of PVBPA-g-PNVP amphiphilic polymer bottlebrush (note: (**i**) X = xanthate moiety; (**ii**) the chain-end extended PNVP from the PVBXPA (X = xanthate moiety) was regarded as a part of grafted chains on the amphiphilic polymer bottlebrush).

**Figure 1:**
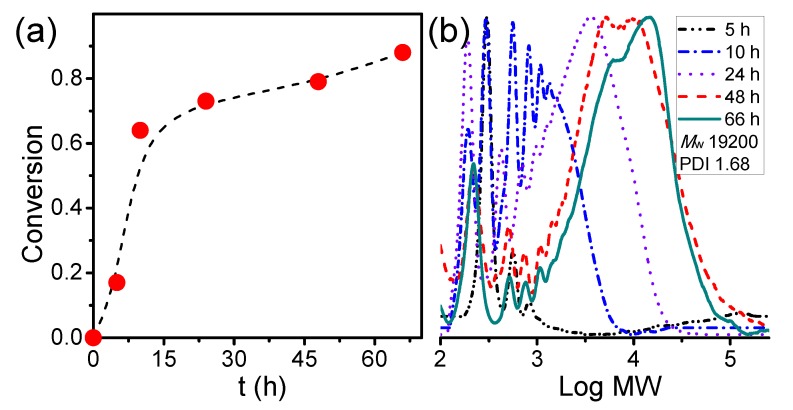
Kinetics of atom transfer radical polyaddition (ATRPA) of 4-vinylbenzyl 2-bromo-2-phenylacetate (VBBPA): (**a**) conversion vs. t and (**b**) evolutions of molecular weights (MW) (VBBPA/CuBr_2_/Cu/ dNBpy = 50/2/1/6 at 40 °C; [VBBPA]_0_ = 1.8 M in anisole).

**Figure 2:**
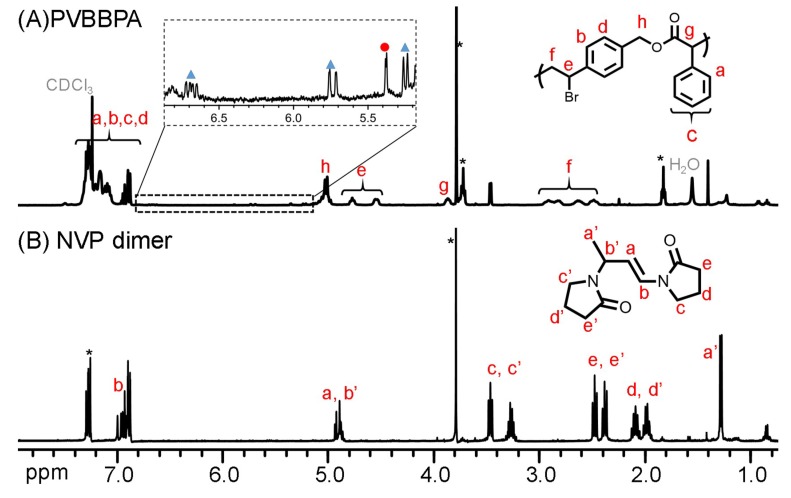
^1^H NMR spectra of (**A**) PVBBPA and (**B**) NVP dimer (* represents solvent peaks of anisole).

**Scheme 2:**
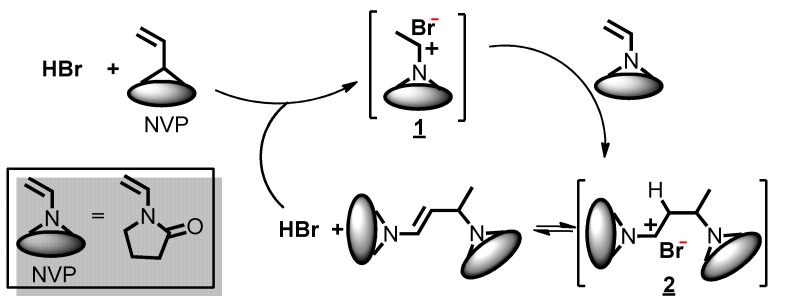
Proposed acid-catalyzed dimerization mechanism during ATRP of PVBBPA with NVP.

To synthesize amphiphilic polymer bottlebrush, as shown in Scheme 1a, we first conducted ATRPA of VBBPA to synthesize hydrophobic polyester backbone. A reagent ratio of VBBPA/CuBr_2_/Cu/L = 50/2/1/6 (L = dNbpy or PMDETA) was employed. Using L = dNbpy, the kinetic results are displayed in Figure 1. Although the conversion of VBBPA increased to over 60% after 10 h, the molecular weight (MW) of the obtaining products showed significant increase after 24 h reaction time. It indicated that the polymerization plausibly proceeded with a step-growth mechanism instead of a chain-growth mechanism, and that we can expect to obtain a linear polyester backbone. Using L = PMDETA, we could obtain any high MW polymers (as shown in Figure S1). It might be ascribed to the loss of high selectivity between the backbone and the chain end initiations. The chemical structure characterization of the obtaining polymer using L = dNbpy was analyzed by ^1^H NMR and FT-IR. Figure 2A shows the ^1^H NMR spectrum of the obtaining PVBBPA. We observed the small signals of double bond chain end (*CH_2_=CH*–Ph–) at ca. 6.7, 5.75, and 5.25 ppm (the triangle symbols in the inserted figure). The lateral bromide in backbone (–CH_2_*CH*(Br)–Ph–) was confirmed as peak e with an average of ca. 4.7 ppm. We observed that the other chain end of bromo-2-phenylacetate (–C(O)O–*CH*(Ph)–Br) was located at ca. 5.4 ppm (the circle symbol in the inserted figure). The other characteristic peaks were also assigned in Figure 2A. From the FT-IR spectrum exhibited in Figure S2a, the PVBBPA characteristic peak of benzene ring at 3030 cm^–1^ and ester group of C=O at 1735 cm^−1^ and C–O at 1150 cm^–1^ was revealed, respectively. These results indicated that a linear type polyester was obtained. The obtaining PVBBPA mainly composes benzylic bromides along the backbone that can be utilized as ATRP initiating sites. To graft hydrophilic side chain, as shown in Scheme 1b, we then conducted ATRP of PVBBPA with NVP (NVP/PVBBPA/CuBr/PMDETA = 200/1/1/1 in anisole at 80 °C). We obtained a high conversion of NVP in less than 2 h. However, we cannot obtain any progress in the MWs from the GPC traces shown in Figure S3. The retention times remained nearly unchanged (ca. 17.7 min). The purified product was analyzed by ^1^H NMR spectroscopy. As shown in Figure 2B, a NVP dimer structure was clearly observed [CDCl_3_, δ =ppm: 6.94–6.98 (–CH=*CH*–N<, 1H), 4.93–4.80 (CH_3_*CHCH*=CH–N<, 2H), 3.47–3.19 ((>N–*CH_2_*CH_2_CH_2_–)×2, 4H), 2.47–2.32 ((>N–CH_2_*CH_2_*CH_2_–)×2, 4H), 2.12–1.90 ((>N–CH_2_CH_2_*CH_2_*-)×2, 4H), 1.28 (*CH_3_*CHCH=CH-N<, 3H)]. Unexpectedly, an acid-catalyzed dimerization of NVP plausibly occurred through a cationic mechanism [58,59]. The acid sources might have resulted from S_N2_ reactions between benzylic bromides and NVP. As proposed in Scheme 2, the benzylic bromide catalyzed dimerization of NVP could start from protonation of a NVP with an HBr to form an intermediate species **1**. Addition of a species **1** and another NVP led to a dimerized intermediate species **2**. Through the resonance of species **2**, a stable structure of NVP dimer was formed and concurrently released HBr to initiate another catalytic cycle. 

**Figure 3:**
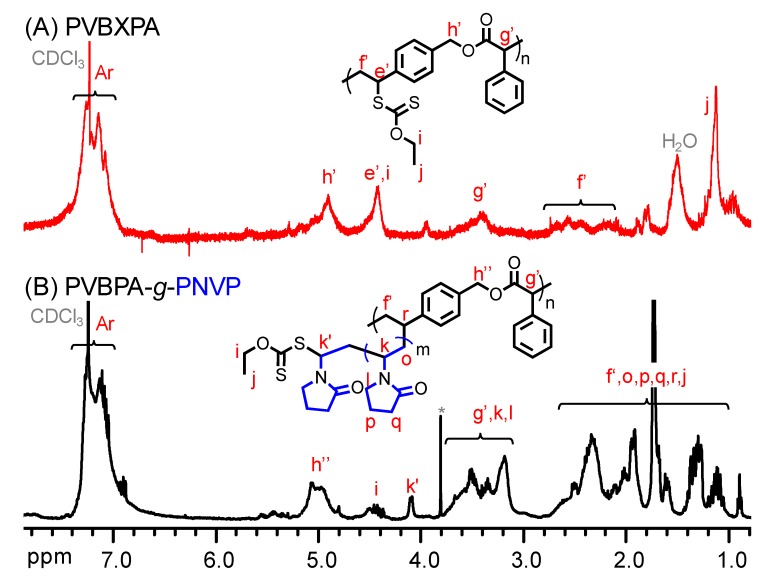
^1^H NMR spectra of (**A**) PVBXPA and (**B**) PVBPA-*g*-PNVP (co)polymers.

**Figure 4:**
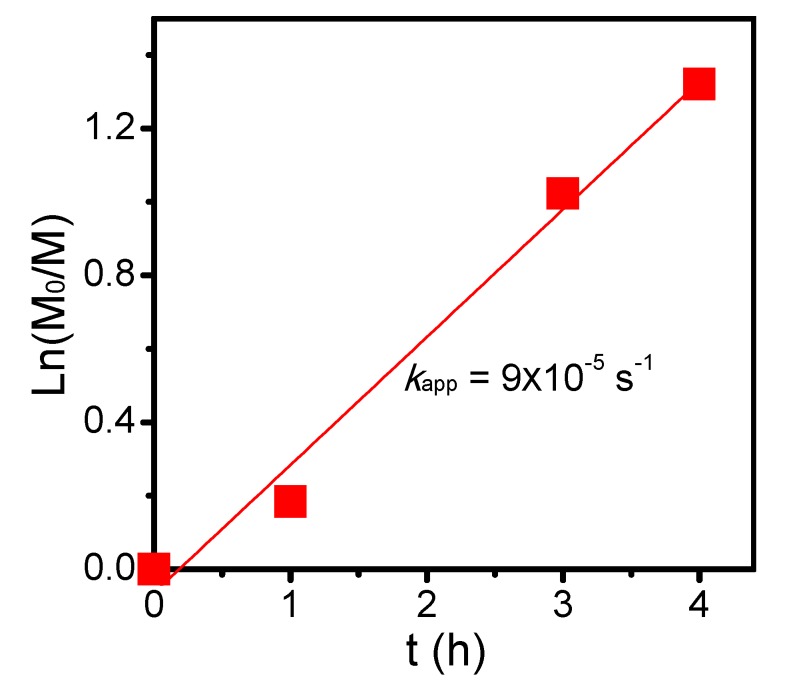
Reversible addition–fragmentation chain transfer (RAFT) polymerization kinetics of PVBXPA with NVP (NVP/PVBXPA/AIBN = 20/1/0.1 at 60 °C; [NVP]_0_ = 4.0 M in anisole).

To alternatively proceed polymerization from the polyester backbone, PVBBPA should be functionalized. Through the S_N_2 reaction shown Scheme 1c, the lateral bromides were replaced by xanthate moieties that were reported as one of the efficient RAFT agents to achieve good control in the polymerization of less-active monomer (herein, NVP) [60]. The PVBBPA polyester was reacted with potassium ethyl xanthate (PEX) to obtain PVBXPA macro-chain transfer agent (macro-CTA). Figure 3A shows the ^1^H NMR spectrum of obtaining PVBXPA. The appearance of peak j (ca. 1.1 ppm) could be attributed to the modified ethyl xanthate moieties (–SC(S)–OCH_2_C*H*_3_). The appearance of peaks e’, h’, and i (ca. 4.8–4.4 ppm) indicated substitution with high conversion. The other characteristic peaks were also revealed in Figure 3A. In FT-IR spectrum, Figure S4a shows a significant increase of intensity at 2930 cm^–1^ representing aliphatic C–H bond from ethyl xanthate moieties. These results illustrated quantitative modification of lateral bromides to ethyl xanthate was successful and obtained PVBXPA macro-CTA. The kinetic of chain extension via RAFT polymerization of PVBXPA macro-CTA with NVP was traced by GC (NVP/PVBXPA/AIBN = 20/1/0.1 at 60 °C; [NVP]_0_ = 4.0 M in anisole). As displayed in Figure 4, a pseudo linear first order reaction was obtained with an apparent reaction rate constant (*k*_app_) of 9 × 10^–5^ s^–1^. After we grafted PNVP, notably, very weak intensities of GPC traces were obtained due to nearly undetectable refractive index differences (i.e., isorefractive between PNVP-based copolymer and THF eluent) [61]. Thus, the copolymer was analyzed by ^1^H NMR spectroscopy. Figure 3B shows the ^1^H NMR spectrum of the corresponding product. The characteristic peaks of g’, h", r, and f’ were from the polyester backbone we detected. The main peaks from PNVP chain were observed in ca. 3.6–3.2 (k and l) and 2.2–1.1 (o, p, and q). Peaks of the PNVP chain end were approximated at ca. 4.5 (i), 4.1 (k’), and 0.8 (j) ppm. Figure S4b demonstrates several clear characteristic peaks of alkyl and amide groups resulting from the grafted PNVP segments. These results illustrated that synthesis of amphiphilic polymer bottlebrushes was successful. Combined with the GPC (i.e., to obtain the n value) and the ^1^H NMR results (i.e., to obtain the m value from the peak ratios of k’, g’, k, and l), we can estimate the overall repeat unit values for PVBPA and PNVP (herein, sample c in Table 1: PVBPA_34_-*g*-PNVP_60_). Thus, we can easily adjust the hydrophobic length by ATRPA and hydrophilic length by RAFT polymerization. The obtained PVBPA_n_-*g*-PNVP_m_ bottlebrushes, including MW and hydrophilic/hydrophobic ratio, are summarized in Table 1. Accordingly, we performed micellization of the PVBPA-*g*-PNVP bottlebrushes in aqueous solution. Critical micelle concentration (CMC) probed by pyrene and particle size estimated by dynamic light scattering (DLS) were also summarized in Table 1. Overall, the CMC values were less than 0.5 mg/mL and the particle sizes were in a range of ca. 100–120 nm. 

### 3.2. Micellization and Cell Uptake Study of the Amphiphilic Polymer Bottlebrush

**Figure 5:**
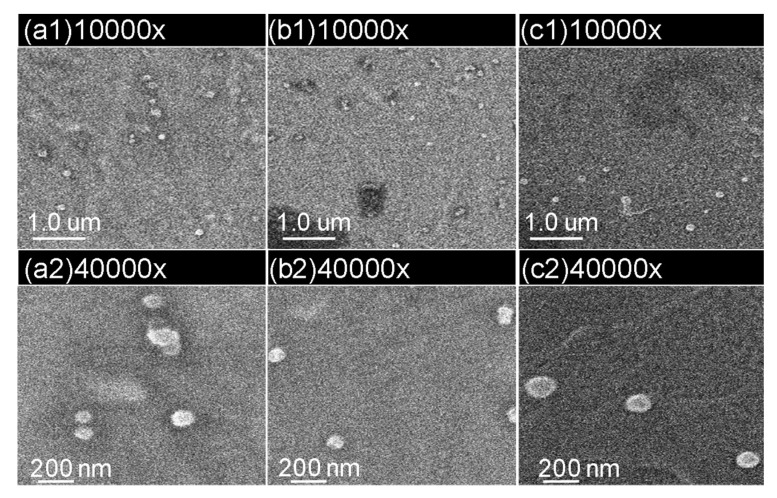
Scanning electron microscopy (SEM) images of micelles prepared from different polymer bottlebrushes (**a**) PVBPA_27_-*g*-PNVP_4_, (**b**) PVBPA_80_-*g*-PNVP_120_, and (**c**) PVBPA_34_-*g*-PNVP_60_.

**Figure 6:**
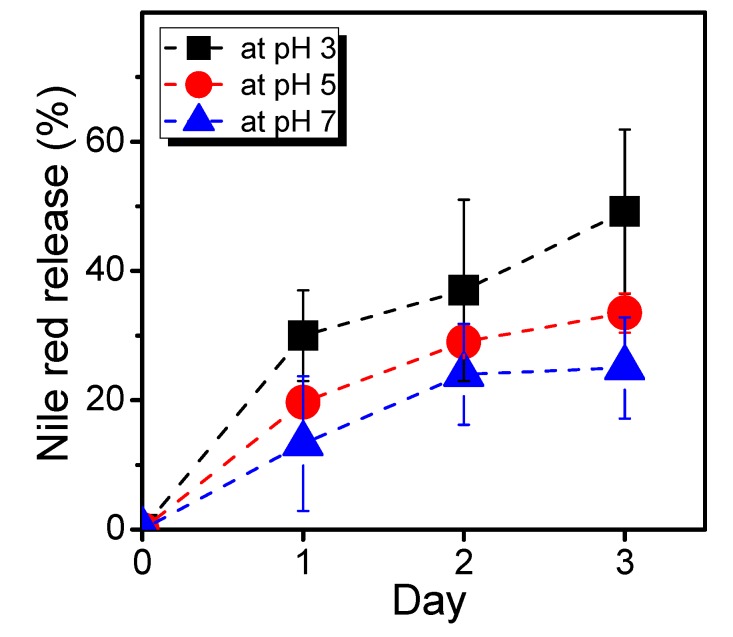
Release profiles of Nile red encapsulated by PVBPA_34_-*g*-PNVP_60_ amphiphilic bottlebrush.

**Figure 7:**
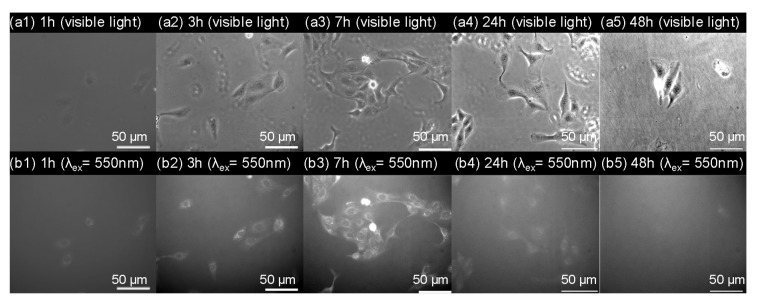
(**a**) Bright field and (**b**) fluorescent images (λ_ex_ = 550 and λ_em_ = 630 nm) of Madin-Darby Canine Kidney (MDCK) cells in the presence of Nile red-containing PVBPA_34_-*g*-PNVP_60_ micelles.

We then conducted scanning electron microscopy (SEM) characterization of three PBVPA-*g*-PNVP amphiphilic polymer bottlebrushes to observe the microstructures of the micelles. PBVPA-*g*-PNVP copolymers were individually dissolved in DMF (1 mg mL^–1^) and slowly added to DI water to form micelles. Each aqueous solution was dialyzed for three days to obtain a stable structure. Prior to SEM measurements, the samples were proceeded with a freeze-dried process to maintain the shape of the microstructures. Figure 5 displays the corresponding SEM images with different magnification (i.e., 10,000× and 40,000×) of the a–c copolymers as listed in Table 1. We observed spherical micelles in each case. The polymer bottlebrushes possess a similar size with an average of ca. 100 nm, which is slightly smaller than the results of DLS. It is rationally ascribed to slight shrinkage after freeze-drying of the samples. Notably, sample c can be easily acquired with a proper amount after purification, compared to samples a and b, which might be due to its good balance of hydrophobic/hydrophilic ratios. For a potential application of nano-carrier systems, we used PBVPA_34_-*g*-PNVP_60_ amphiphilic polymer bottlebrush to examine the drug release profiles of a model drug of Nile red. A mixture of 20 mg of the PVBPA_34_-g-PNVP_60_, 2 mg of Nile red, and 1 mL of DMF were well-mixed. The solution was slowly added to 4 mL of deionized water and dialyzed in a 50 mM PBS solution at pH = 7 to remove free Nile red. The neutral solutions were separately kept in dialysis bags and immersed in PBS solution with different pH (7, 5, and 3). The release of Nile red was traced by fluorescence spectroscopy. Figure 6 displays the Nile red release profiles at different pH environments. At pH 7, slow release was observed, and the maximum release percentage was approximated to 25% in three days. At pH 5, we detected a gradual increase up to approximately 35% of the Nile red in three days. At pH 3, gradual increase and release up to approximately 50% were detected. These results illustrated that the micelles had a structurally insignificant change in a neutral environment but might be disrupted in an acidic environment which should be ascribed to the degradation of ester groups along the hydrophobic backbone. Plausibly, the gradual release profiles at pH 3 and 5 might be due to slow cleavage rate of the PVBPA polyester backbone. We observed insignificant burst release profiles in the initial periods. We eventually examined the cytotoxicity and cell uptake of the polymer bottlebrush micelles. A 24-well culture dish with 5000 MDCK cells in each well was prepared. After the cells stabilized, a micelle solution of PVBPA_34_-g-PNVP_60_ (4 mg/mL) containing Nile red was individually added into the wells. Figure 7 presents the MDCK cell images with different incubation times observed under visible light and excitation at 550 nm (magnification: 40X). As shown in Figure 7a1–a5, we observed the preservation of the elongated shape of MDCK cells, indicating a low cytotoxicity during the incubation time. As shown in Figure 7b1–b5, interestingly, we detected significant cell uptake in 7 h based on the increase of the Nile red fluorescent intensity. Incubated after 7 h, we observed a gradual disappearance of the dye fluorescent intensity. These results illustrated that the polymer bottlebrush micelles can be taken up through the endocytosis of MDCK cells. Later on, the Nile red-containing micelles were excluded out from the MDCK cells through a regular metabolic mechanism as normal cells possess an intracellularly neutral environment. The results showed consistent trends to the release profiles in Figure 6. 

## 4. Conclusions 

Through a step-growth mechanism, we obtained a linear type PVBBPA polyester with benzylic bromides along the backbone. Using ATRP for chain extension, NVP dimers were quantitatively obtained. The PVBBPA backbone might provide acid sources to catalyze the dimerization of NVP. Alternatively, the lateral bromides of PVBBPA can be quantitatively replaced by xanthate moieties to obtain PVBXPA which can serve as a macro-CTA. Using RAFT polymerization for chain extension, we observed a pseudo linear first order reaction with a *k*_app_ of 9 × 10^–5^ s^–1^. Thus, several PVBPA_n_-*g*-PNVP_m_ bottlebrushes were synthesized and characterized by ^1^H NMR and FT-IR spectroscopy. The CMCs of the bottlebrushes were less than 0.5 mg/mL (probed by pyrene) and the particle sizes were in a range of ca. 100–120 nm (measured by DLS). Compared the results of SEM and DLS, similar particle sizes were obtained. For a potential application of nano-carrier systems, encapsulations of Nile red by PVBPA_34_-g-PNVP_60_ were conducted and release of Nile red was traced by fluorescence spectroscopy. Slow release and low efficiency were observed at pH 7. Faster releases and higher efficiencies were acquired at pH 5 and 3 compared to the trends at pH 7. Eventually, cytotoxicity and MDCK cell uptake were examined in the presence of Nile red-containing PVBPA_34_-g-PNVP_60_ micelles. From the apparent images of optical microscopy (OM), the elongated shape of the MDCK cells was preserved, illustrating a low cytotoxicity during the incubation time. By tracing the fluorescent images during the incubations, interestingly, we detected significant cell uptake in 7 h. Thereafter, a gradual disappearance of the fluorescent intensity was observed. It is plausible that the Nile red-containing micelles can be taken up and then excluded out from the MDCK cells through a regular metabolic mechanism as normal cells possess an intracellularly neutral environment. We provided an alternative strategy to the preparation of amphiphilic polymer bottlebrush that forms the proper size for the endocytosis of MDCK cells. Further examinations on the application of anticancer cells are currently under way. 

## Figures and Tables

**Table 1:** Characterization of PVBPA_n_-*g*-PNVP_m_ bottlebrushes.

Sample	n Units of VBBPA	m Units of NVP	Hydrophobic/Hydro-philic Ratio ^1^	CMC (mg/mL) ^2^	Particle Size (nm) ^3^
(a)	27	4	95/5	0.39	120
(b)	80	120	50/50	0.28	102
(c)	34	60	42/58	0.49	96

^1^ Ratio was defined as *M*_n,PNVP_/*M*_n,PVBPA_. ^2^ CMC was probed by pyrene compound based on the slope variation of the fluorescence intensity. ^3^ Particle size was estimated by DLS based on weighted average diameter with a concentration of 1 mg/mL H_2_O.

## Supplementary Materials

The following are available online at https://www.mdpi.com/2073-4360/11/6/1079/s1, Figure S1: GPC traces for ATRPA of VBBPA (VBBPA/CuBr_2_/Cu/PMDETA), Figure S2: FT-IR spectra of (**a**) PVBBPA and (**b**) purified NVP dimer obtained after ATRP (NVP/PVBBPA/CuBr/PMDETA), Figure S3: GPC traces for ATRP of PVBBPA with NVP at various reaction times (NVP/PVBBPA/CuBr/PMDETA), Figure S4: FT-IR spectra of (**a**) PVBXPA and (**b**) PVBPA-*g*-PNVP (co)polymers.
