# Supplementary material for: Synthesis of Poly(N-vinylpyrrolidone)-Based Polymer Bottlebrushes by ATRPA and RAFT Polymerization: Toward Drug Delivery Application
Source: Polymers (Basel). 2019 Jun 22;11(6):1079. doi: 10.3390/polym11061079 (PMC6631111; doi:10.3390/polym11061079)
Supplement: Supplementary file 1 [file polymers-11-01079-s001.pdf]

Supporting Information for

Synthesis of Poly(*N*-vinylpyrrolidone)-based Polymer Bottlebrushes by ATRPA and RAFT Polymerization: Toward Drug Delivery Application

Yi-Shen Huang[1], Jem-Kun Chen[2], Shiao-Wei Kuo[3], Ya-An Hsieh[1], Shota Yamamoto[4], Jun Nakanishi[4,*] and Chih-Feng Huang[1,*]

[1] Department of Chemical Engineering, National Chung Hsing University, 145 Xingda Road, Taichung 40227, Taiwan; yishen617@gmail.com (Y.-S.H.); yaan_hsieh@yahoo.com.tw (Y.-A.H.); HuangCF@dragon.nchu.edu.tw (C.-F.H.)

[2] Department of Materials Science and Engineering, National Taiwan University of Science and Technology, Taipei 10607, Taiwan; jkchen@mail.ntust.edu.tw (J.-K.C.)

[3] Department of Medicinal and Applied Chemistry, Kaohsiung Medical University, Kaohsiung 807, Taiwan; kuosw@faculty.nsysu.edu.tw (S.-W.K.)

[4] World Premier International Research Center for Materials Nanoarchitectonics (WPI-MANA), National Institute for Materials Science (NIMS), 1-1, Namiki, Tsukuba, Ibaraki 305-0044, Japan; YAMAMOTO.shato@nims.go.jp (S.Y.); NAKANISHI.Jun@nims.go.jp (J.N.)

Captions.

Figure S1. GPC traces for ATRPA of VBBPA (VBBPA/$CuBr_2$/Cu/PMDETA = 50/2/1/6 at 40 °C; $[VBBPA]_0$ = 1.8 M in anisole).

Figure S2. FT-IR spectra of (a) PVBBPA and (b) purified NVP dimer obtained after ATRP (NVP/PVBBPA/CuBr/PMDETA = 200/1/1/1 in anisole; PVBBPA: M_n = 11200 and PDI = 1.58; $[NVP]_0$ = 4.0 M).

Figure S3. GPC traces for ATRP of PVBBPA with NVP at various reaction times (NVP/PVBBPA/CuBr/PMDETA = 200/1/1/1 in anisole at 80 °C).

Figure S4. FT-IR spectra of (a) PVBXPA and (b) PVBPA-*g*-PNVP (co)polymers.

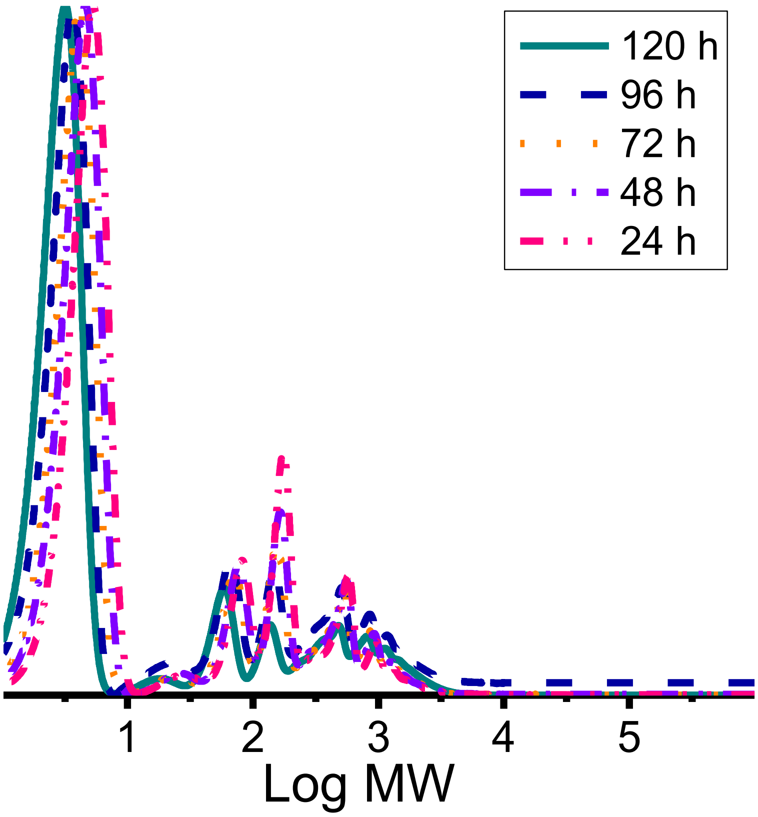

Figure S1. GPC traces for ATRPA of VBBPA (VBBPA/$CuBr_2$/Cu/PMDETA = 50/2/1/6 at 40 °C; $[VBBPA]_0$ = 1.8 M in anisole).

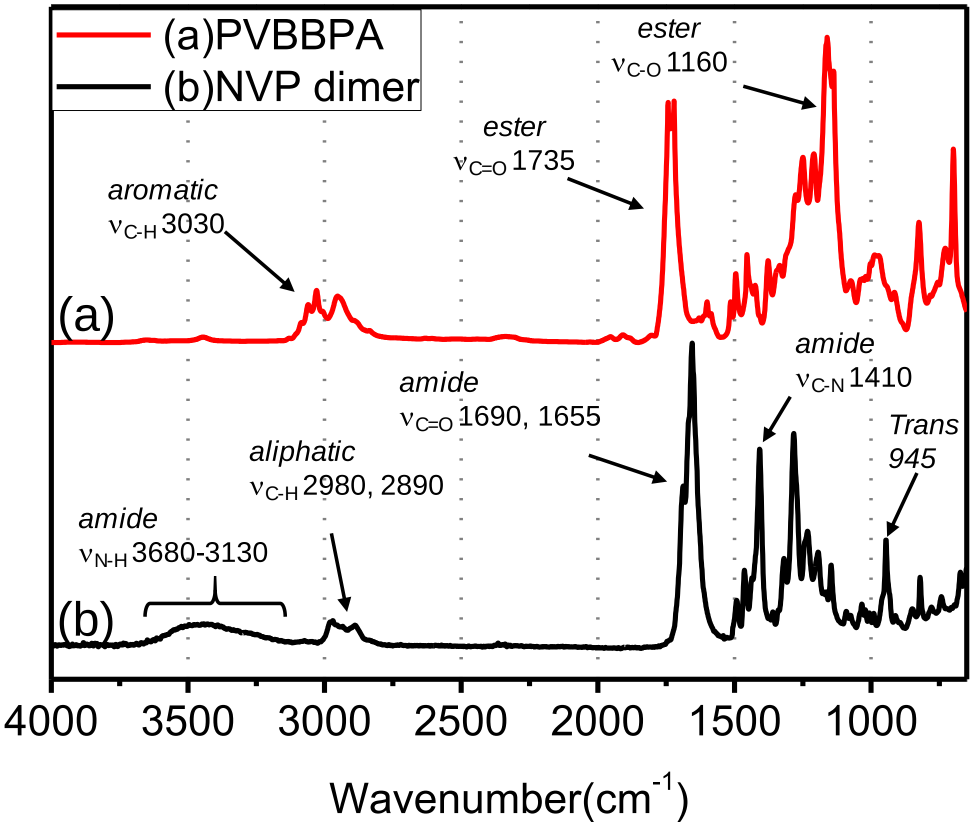

Figure S2. FT-IR spectra of (a) PVBBPA and (b) purified NVP dimer obtained after ATRP (NVP/PVBBPA/CuBr/PMDETA = 200/1/1/1 in anisole; PVBBPA: M_n = 11200 and PDI = 1.58; $[NVP]_0$ = 4.0 M).

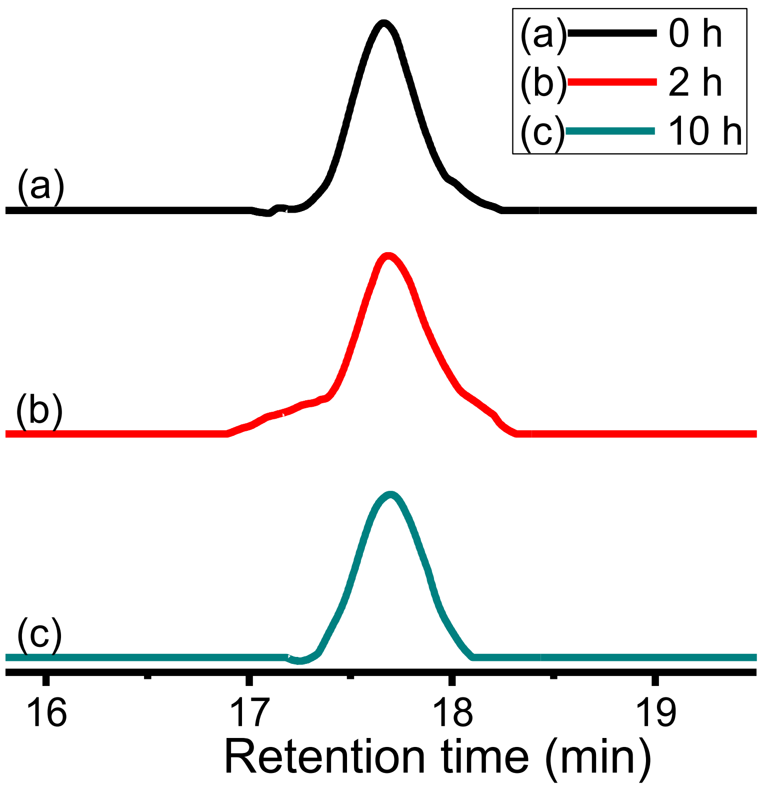

Figure S3. GPC traces for ATRP of PVBBPA with NVP at various reaction times (NVP/PVBBPA/CuBr/PMDETA = 200/1/1/1 in anisole at 80 °C).

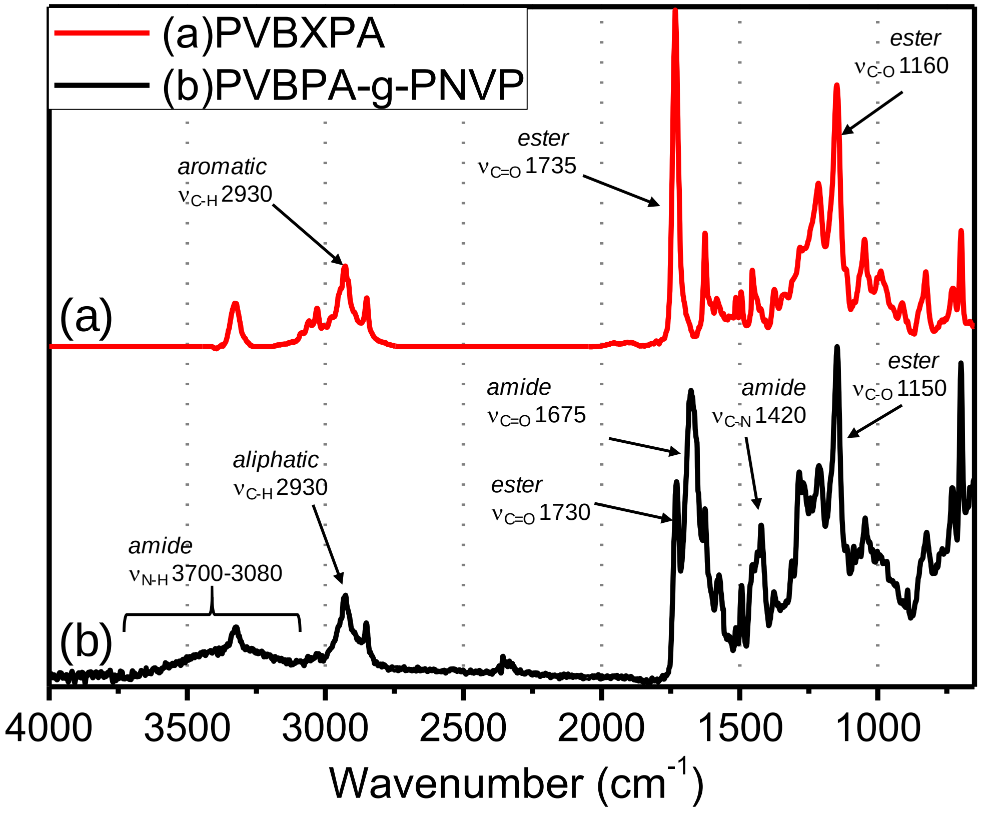

Figure S4. FT-IR spectra of (a) PVBXPA and (b) PVBPA-*g*-PNVP (co)polymers.
